# Supplementary material for: WordCommentsAnalyzer: A windows software tool for qualitative research
Source: F1000Res. 2018 Sep 4;7:536. Originally published 2018 May 3. [Version 2] doi: 10.12688/f1000research.14819.2 (PMC6137411; doi:10.12688/f1000research.14819.2)
Supplement: Supplementary file 3 [file f1000research-7-17614-s0002.tgz › b2a79496-4ada-47c4-9682-a99ce867836d.zip!/Eight Iranian Health Journals/Archives of Rehabilitation_67.docx]

© Archives of Rehabilitation. This work is licensed under CC BY-NC 4.0 (https://creativecommons.org/licenses/by-nc/4.0/).

Volume & Issue: دوره 3 - شماره 3

Title: کاربرد نقاشی برای کاهش اضطراب بیماران اسکیزوفرنیک

http://rehabilitationj.uswr.ac.ir/article-1-567-fa.html

هدف: بیماران اسکیزوفرنیک نوع مداوم که بیشتر به عنوان مزمن خوانده می شدند بخش عمده ای از بیماران بستری در بخش های توان بخشی را تشکیل می دهند. به جز نشانه های خاص اسکیزوفرنیا ، این بیماران عموما با اضطراب نیز روبرو هستند. این اضطراب که عموما با داروهای ضدروان پریشی برطرف نمی شود ممکن است بخشی از طبیعت اسکیزوفرنیا باشد اما گاهی می تواند ناشی از ساختار و کیفیت ویژه ی زندگی این بیماران در بخش های بیمارستانی باشد. هر کوششی در جهت کاهش اضطراب این بیماران را می توان گامی به سوی درمان و توان بخشی کامل تر و بهبود کیفیت زندگی آنان دانست. در این پژوهش کاربرد نقاشی در کاهش اضطراب این بیماران بررسی شده است. روش بررسی: 100 بیمار اسکیزوفرنیک با طول دوره بیماری حداقل دو سال بر مبنای DSM-IV در بخش های توان بخشی دراز مدت مرکز روان پزشکی رازی به صورت تصادفی انتخاب شدند. میزان اضطراب آنان از طریق آزمون اضطراب کتل سنجیده شد. سپس 50 نفر از آنان به عنوان گروه تجربی انتخاب شدند که به مدت 15 جلسه هر بار یک ساعت به طراحی و نقاشی پرداختند و در همان ساعات به فعالیت هایی که جنبه ی خنثی داشت پرداختند. داروهای بیماران در طول مدت پژوهش تغییر داده نشد. در پایان 15 جلسه مجددا میزان اضطراب بیماران سنجیده شد. نمره های بار اول و دوم گروه تجربی و نمره ی بار دوم گروه تجربی و گواه به وسیله ی آزمون آماری t-student با یکدیگر مقایسه شد. یافته‌ها: در مقایسه ی میانگین نمره های اضطراب حاصل آزمون کتل با استفاده از روش t-student معلوم شد که کاربرد نقاشی در حد معنی داری از نظر آماری سبب کاهش اضطراب بیماران اسکیزوفرنیک می شود. (t=67/5 و p<0.01) نتیجه‌گیری: جلسات طراحی و نقاشی با تعداد و دوره های متناسب می تواند به طور چشمگیری اضطراب بیماران اسکیزوفرنیکی که مدت زمان زیادی است مبتلا به اسکیزوفرنی هستند در بخش توانبخشی کاهش دهد.

Title: بررسی مهارت های ارتباطی و تعاملی در بیماران روان‌پریش

http://rehabilitationj.uswr.ac.ir/article-1-568-fa.html

هدف: این تحقیق با هدف بررسی مهارت های ارتباطی و تعاملی، بر روی بیماران روان پریش بالای 18 سال در سال 1379، انجام گرفت. روش بررسی: نمونه ی مورد مطالعه شامل 80بیمار روان پریش ( 38زن و 42مرد) از 4 مرکز درمانی رعد، سینا، رازی، امام حسین (ع) بودند که روش مطالعه, توصیفی – مقطعی بود و نمونه های مورد مطالعه به صورت غیر احتمالی و با روش نمونه گیری متناسب با اندازه، انتخاب شدند. ابراز مورد استفاده جهت گردآوری اطلاعات، آزمون مشاهده ای ACISبود که اطلاعات آن توسط کاردرمانگر تکمیل شده است. آزمونهای آماری مورد استفاده در این مطالعه عبارت است از: ضریب همبستگی اسپیرمن، ویل کاکسون و من ویتنی. یافته‌ها: میانگین رتبه آزمون ACIS در گروه بیماران اسکیزوفرنی ، به صورت معنی داری پایین تر از گروه بیماران اسکیزوافکتیو بود(P<0/001) میانگین رتبه آزمون (ACIS) در گروه بیماران بستری به صورت معنی داری پایین تر از گروه بیماران سرپایی بود(P<0/001) میانگین رتبه ی آزمون ACIS در گروه بیماران روان پریش زن و مرد تفاوت معنی داری نشان نداد.  نتیجه گیری: نتایج به دست آمده ضعف مهارت های ارتباطی و تعاملی در بیماران اسکیزوفرنی و نیز بیماران بستری را مشخص کرد. نتایج این تحقیق اطلاعات مفیدی در رابطه با مهارت های ارتباطی و تعاملی در اختیار کاردرمان گرانی که با بیماران سروکار دارند ، قرار میدهد. لیکن شناخت دقبق تر این مهارت ها در اختلال و مقعیت های دیگر ، احتیاج به بررسی بیش تری دارد.

Title: بررسی فراوانی علائم مثبت و منفی در بیماران مزمن اسکیزوفرنیک زن

http://rehabilitationj.uswr.ac.ir/article-1-569-fa.html

هدف:‌ بیماری اسکیزوفرنی، با آن که به عنوان یک بیماری واحد تحت بررسی قرار می گیرد ولی شامل یک سری اختلالاتی است که در یک  گروه نامتجانس می باشند. بیماران به تیپ 1,2 تقسیم می شوند. بیماران تیپ 1 علائم مثبت و 2 علائم منفی بارزی را دارا هستند. روش بررسی: گروه مورد بررسی 50 زن با تشخیص اسکیزوفرنی مزمن سال ها تحت درمان با حداقل یک نوع داروی ضد سایکوز بوده اند. با استفاده از چک لیست، علائم منفی و مثبت ارزیابی گردید. یافته‌ها:‌ در بیماران مزمن توهمات (از علائم مثبت) در کنار اکثر علائم منفی دیده می شود. به بیان دیگر، علائم منفی بیمار را بیش تر تخریب نموده و کارآیی وی را مختل می سازد. در بین علائم منفی، کندی یا سطحی شدن عاطفه – فقدان انگیزش بی توجهی اجتماعی و عدم توانایی در صمیمی شدن، بیش تر از بقیه یافت می گردد. نتیجه‌گیری: بیمارانی که به داروهای ضد سایکوز کنونی جواب مناسب را نداده اند باید مورد بررسی بیش تر قرار گرفته و در درمان زن ها از داروهای جدیدتری استفاده نمود.

Title: ویژگی‌های روان‌سنجانه مقیاس رضایت از خدمت ۳۰ در جمعیت ایرانی

http://rehabilitationj.uswr.ac.ir/article-1-570-fa.html

هدف: با توجه به گسترش مراکز ارایه دهنده ی خدمات مشاوره در سال های اخیر، ارزشیابی آنها ضرورت مبرم پیدا کرده و یکی از مهمترین اجزای این ارزشیابی، سنجش رضایت خدمت گیرندگان مراکز یاد شده است. بر این اساس، پرسشنامه ی سی سوالی "رضایت خدمت"به فارسی برگردانده و ویژگی های روان سنجانه ی آن در جمعیت ایرانی بررسی شد. این مقیاس که محصول برنامه ی رضایت پژوهشی دانشگاه کالیفرنیای سانفرانسیسکو (UCSF) است. روش بررسی: پس از ترجمه به فارسی برای ارزیابی از نظر قابل فهم بودن در جمعیت ایرانی بر روی 33 مراجعه کننده 9 مرکز مشاوره تهران آزموده و اصلاح شد. سپس برای سنجش پایانی آن، بر روی 121 مراجعه کننده (%71 زن و%29مرد با دامنه سنی 18-65سال) و برای تعیین روایی آن، بر روی 50 مراجعه کننده (%78زن و %22 مرد با دامنه سین 18-75) سال اجرا شد. یافته‌ها: میانگین هم بستگی پاسخ به سوال های سیگانه مقیاس (0/35) و ضریب a کرونباخ (0/94) حاکی از هم سانی درونی مقیاس است. روایی ملاکی از طریق محاسبه ی ضریب هم بستگی بین نمرات مقیاس و عملکرد فرد روی سه سوال باز پاسخ انتهای مقیاس تعیین شده (r=0/53). نتیجه گیری: نمره 95 روی مقیاس به عنوان نقطه ی برش مناسب شناخته شد.

Title: بررسی عوامل مؤثر بر طول مدت بستری بیماران روانی در مرکز روان‌پزشکی رازی

http://rehabilitationj.uswr.ac.ir/article-1-571-fa.html

هدف: مساله مراقبت و درمان بیماران روانی یکی از مسائل و معضلات مهم بهداشتی جوامع می باشد. به منظور بررسی عوامل موثر بر طول مدت بستری بیماران روانی مطالعه ای بر روی بیماران بستری شده در مرکز روان پزشکی رازی در طی نیم سال دوم سال 79 انجام شد. روش بررسی: این مطالعه، به روش گذشته نگر – توصیفی انجام گرفته است. یافته‌ها: نتایج حاصل از این تحقیق نشان داد که بین عواملی چون سن و جنس بیماران، محل تولد، محل سکونت، وضعیت اشتغال، منبع ارجاع و نوع درمان بیماران با طول مدت بستری رابطه ای وجود نداشت.  نتیجه‌گیری: این مطالعه نشان داد که بیماران مبتلا به اسکیزوفرنیا نسبت به بیماران خلقی و سایر بیماران زمان اقامت طولانی تری را در بیمارستان داشتند (P<0/005). هم چنین بیماران با دفعات بستری قبلی، بیماران با طول مدت اختلال طولانی تر، بیماران با داشتن بیماری همراه (اعم از جسمی یا روانپزشکی)، بیماران مجرد و طلاق گرفته نسبت به بیماران متاهل و بیماران با پوشش بیمه، زمان اقامت طولانی تری را در بیمارستان داشته اند.

Title: بررسی فراوانی علائم منفی در یک صد بیمار بستری مبتلا به اسکیزوفرنیا در مرکز آموزشی درمانی روان‌پزشکی رازی

http://rehabilitationj.uswr.ac.ir/article-1-572-fa.html

هدف: بروز علائم منفی در اسکیزوفرنیا از جمله اصلی ترین مشکلات موجود بر سر راه توان بخشی این دسته از بیماران محسوب می گردد. استفاده از داروهای مناسب جهت کاهش این علائم و انجام درمان های روانی – اجتماعی به خصوص آموزش مهارت های اجتماعی در هم گامی با سایر امکانات و تسهیلات روان پزشکی جامعه نگر نقش کلیدی را در حد این معضل ایفا می نماید. در مقاله ی حاضر پس از بحث و شناخت دقیق تری در خصوص علائم منفی و درمان های متداول آنان، به بررسی فراوانی این علائم در یک صد بیمار اسکیزوفرنیک بستری در مرکز روان پزشکی رازی و ارتباط سه متغیر سن، جنس و ازمان بیماری با این علائم می پردازیم.  روش بررسی: یک درصد بیمار اسکیزوفرنیک بستری در مرکز روان پزشکی رازی به طور تصافی و با نسبت مساوی از بین زنان و مردان از بخش های حاد و نگه داری انتخاب و با پرسش نامه ی SANS نسبت به ثبت علائم منفی در آنان اقدام گردید. سپس با استفاده از روش های آماری، ارتباط این یافته ها با متغیرهای یادشده، مورد آزمون قرار گرفت. یافته ها: گستردگی علائم منفی بیماران از %86 تا %98 علائم شدید نیز از %30 تا %55 در نوسان بود. بنابراین هیچ بیماری بدون علامت منفی نبود. ایم این آمار هم از حیث مقدار و هم از حیث شدت به طور مشخص بیش از آمار مندرج در کتاب روان پزشکی کاپلان و سادک است. شدیدترین علائم منفی در بخش نگه داری زنان و خفیف ترین آن نیز در بخش حاد زنان ملاحظه گردید. هیچ گونه ارتباط معنی داری بین شدت علائم منفی و متغیرهای سن، جنس و مدت ابتلاء به بیماری یافت نشد. نتیجه‌گیری: گستردگی فراوانی علائم منفی در نزد بیماران اسکیزوفرنیک این مرکز و شدت آن واقعیتی است که بیانگر وخامت اوضاع کلی این دسته از بیماران و مستلزم به کارگیری شیوه های مناسب درمانی جهت تقلیل این علائم و افزایش آموزش پذیری آنان برای زندگی مستقل تر در بستر اجتماع و خانواده می باشد.

Title: بررسی مقایسه‌ای نحوه مراجعه به مطب روان‌پ‍زشکی در تهران و یک شهرستان

http://rehabilitationj.uswr.ac.ir/article-1-573-fa.html

هدف: پژوهش حاضر مطالعه ای توصیفی و تحلیلی است که به منظور بررسی و شناخت مسیر مراجعه به روان پزشک در شهرستانی با تجربه ی جدید یک روان پزشک در مقایسه به تهران صورت گرفته است. متغیرهای مورد مطالعه شامل مشخصات جمعیت شناختی مراجعان، منبع ارجاع، محل مراجعه ی قبلی، استفاده از درمان های سنتی، محلی و نیز زمان بین بروز مشکل یا نشانه ی بیماری تا نخستین مراجعه به روان پزشکی بوده است.  روش بررسی: ابتدا از طریق نمونه گیری تصادفی تعداد هزار نفر مراجعه کننده بار اول به اولین و تنها مطب روانپزشکی در یکی از شهرستانهای استان کرمان به نام سیرجان با جمعیتی بالغ بر یک صد و بیست هزار نفر در سال اول افتتاح مطب و سپس هزار نفر دیگر در طی یک سال بعد انتخاب شدند. در تهران نیز با همین روش تعداد پانصد مراجعه کننده ی جدید برای سال اول تاسیس مطب و تعداد پانصد نفر دیگر نیز برای سال دوم انتخاب شدند. بعد از ارزیابی پرونده ها تمامی مراجعان، متغیرهای پژوهش با استفاده از نرم افزار مربوطه مورد تجزیه و تحلیل آماری قرار گرفت. یافته ها: بیش ترین منبع ارجاع به روان پزشک در هر چهار سال مورد ارزیابی، بستگان و آشنایان مراجعه کنندگان بودند. پزشکان عمومی نقش کمی در ارجاع بیماران به روان پزشک داشتند. در شهرستان سیرجان در طی سال دوم به تدریج میزان ارجاع از طرف متخصصان، پرسنل بهداشتی درمانی و مراکز غیر پزشکی با تفاوت معنادار آماری افزایش یافت. با این حال در تهران تفاوت بین منابع ارجاع در سال اول و دوم معنادار نبود. به ترتیب %33/4 و %8/6 آنها به مطب سیرجان و تهران حداقل یک بار در طول عمر خود به درمانگر محلی مراجعه کرده بودند که از نظر آماری تفاوت معناداری داشت.  نتیجه گیری: تغییرات یاد شده در متغیرهای مورد ارزیابی و مقایسه ی آماری آن در دو شهرستان مورد مطالعه علاوه بر نشان دادن مشخصات جمعیت شناختی و سطح آگاهی مردم در خصوص چگونگی استفاده از خدمات بهداشت روانی می تواند نشان دهنده نقش مداخله های گوناگون آموزشی در سطح کلی جامعه و نیز در سطوح مختلف درمانی برای پزشکان و پرسنل بهداشتی و درمانی دیگر جهت تشخیص صحیح مشکل و ارجاع به موقع بیماران به روانپزشک باشد و بر آن تاکید ورزد.

Title: نیم‌رخ روانی–اجتماعی معتادان نوجوان ایرانی

http://rehabilitationj.uswr.ac.ir/article-1-574-fa.html

هدف: این پژوهش با هدف بررسی برخی مشخصات معتادان نوجوان (y<20) ایرانی شامل جنسیت، سن شروع، نوع ماده ی مصرفی، روش مصرف، سابقه ترک، وضعیت خانوادگی، وضعیت اجتماعی-اقتصادی، وجود علائم روانی، سابقه اقدام به خودکشی، و علت گفته شده برای مصرف انجام شده است. روش بررسی: در این پژوهش از میان بیش از 500مراجعه به یک مرکز سرپایی ترک اعتیاد در تهران، 65 نفر که حداکثر 20 سال سن داشتند و مشخصات آن ها از پرونده ها استخراج شد و به شکل توصیفی وگذشته نگر مورد بررسی قرار گرفتند. که از بین این تعداد %98/46 مذکر بودند. کم ترین سن شروع 8سالگی، بیش ترین ماده ی مورد مصرف تریاک و هروئین (%79/99) و بالاترین روش مصرف "تدخین" (%43/18در مورد تریاک و %57/14 برای هروئین )بود. یافته‌ها: در %76/93 این افراد، حداقل یک بار سابقه ی ترک وجود داشت. %73/84 وضعیت ناهنجار اجتماعی-اقتصادی و %60/00وضعیت ناهنجار خانوادگی از نظرعاطفی داشتند. در تمام این افراد علائم روانی (بیش از همه اضطراب و افسردگی) و در %21/53 از آنها سابقه ی مراجعه به مراکز سرپایی یا بستری روان پزشکی به دلیل علائم روانی وجود داشت، که از میان عده ای اخیر در %28/57 موارد سابقه اقدام به خودکشی بیان شده است. بیشترین علت ادراک شده یا تصوری برای شروع مصرف، وجود مشکلات عاطفی و خانوادگی ( %61/53) ذکر شده بود. نتیجه‌گیری: الگوی مصرف نوجوانان ایرانی علاوه بر دشواری‌هایی که برای ترک دارد، خطر انتقال HIV، هپاتیت و ارتکاب جرم را نیز به همراه دارد. بنابراین ضمن برنامه‌ریزی برای توسعه خدمات پیشگیرانه و درمانی، با توجه به دوره سن نوجوانی که متضمن فعالیت جنسی است، از یک سو وناموفق بودن نسبی درمان‌های مبتنی بر پرهیز کامل از سوی دیگر، باید برای توسعه خدمات و برنامه‌های کاهش عارض نظیر درمان نگه‌دارنده با متادون، تعویض سرنگ، آموزش روابط جنسی سالم، و مشاوره ایدز اقدام‌هایی عاجل به عمل آورد.

Title: رویکردهای اساسی در تدوین استراتژی‌های پیشگیری از مشکلات و آسیب‌های روانی-اجتماعی

http://rehabilitationj.uswr.ac.ir/article-1-575-fa.html

به نظر می آید نتوانیم مبانی تدوین راهبردهای مقابله با آسیب ها و مشکلات روانی اجتماعی را بر اساس نظراتی مشخص، عینی و هم آهنگ و دریک راستای کلی، تدوین کنیم، نخواهیم توانست به راهبردهای بنیادینی دست یابیم که بتواند راهنمای برنامه های مداخل گرانه شده و در پایان نیز مبنای ارزیابی های ما قرار گیرد. در این مقاله، کوشش شده است خطوط اصلی دو رویکرد و به ویژه راهبردهایی که به منظور کاهش مشکلات و آسیب ها ی اجتماعی به کارگرفته شده اند، مورد کاووش قرار گیرد که علاوه بر ابهام و سردرگمی در مبانی مورد نظر، آن چه انجام شده، نه دریک زمینه ی اجتماعی شهرنشینی و بنابر واقعیت جامعه ایران، بلکه بر مبنای این فرضیه عمل کرده ایم، که گویا با یک جامعه ی روستایی روبه رو هستیم، و لذا نتوانسته ایم به نتایج دلخواه تائل آییم. موضوعی که حداقل در ابتدای راه تدوی برنامه چهارم توسعه، حتما باید به آن پرداخته شود.
